# Characterization of plant growth-promoting rhizobacterial isolates associated with food plants in South Africa

**DOI:** 10.1007/s10482-021-01633-4

**Published:** 2021-08-13

**Authors:** Oluwaseun Adeyinka Fasusi, Adenike Eunice Amoo, Olubukola Oluranti Babalola

**Affiliations:** Food Security and Safety Niche, Faculty of Natural and Agricultural Science, North-West University, Private Mail Bag X2046, Mmabatho, 2735 South Africa

**Keywords:** 16S rRNA gene, Bioinformatics, Antifungal activity, Beneficial bacterial, Microbial inoculant, Plant growth

## Abstract

The region around the plant root referred to as the rhizosphere, is the zone where various microbial activity occurs. It performs crucial functions such as increasing the uptake of nutrients for plant development and preventing plant against plant pathogens. Keeping in mind the beneficial role performed by rhizospheric microorganisms, rhizobacterial species were isolated from the maize and soybean plant's rhizosphere. The isolated microorganisms were evaluated for their biochemical characteristics, plant growth-promoting potentials, tolerance to different environmental conditions, and their antifungal activity against *Fusarium graminearum,* a fungal pathogen that infects maize. The rhizobacterial isolates with multiple plant growth-promoting potentials were identified as *Bacillus* spp (80.77%), *Rhodocyclaceae* bacterium (3.85%), *Enterococcus* spp (3.85%). *Massilia* spp (3.85%*.* and *Pseudomonas* (7.69%) species based on their 16S rRNA molecular characterization. The bacterial isolates possessed antifungal activities against *Fusarium graminearum,* promote maize and soybeans seed under laboratory conditions, and exhibited different levels of tolerance to pH, temperature, salt, and heavy metal. Based on this, the whole genome sequencing of *Bacillus* sp. OA1, *Pseudomonas rhizosphaerea* OA2, and *Pseudomonas* sp. OA3 was performed using Miseq Illumina system to determine the functional genes and secondary metabolites responsible for their plant growth-promoting potential Thus, the result of this research revealed that the selected bacterial isolates possess plant growth-promoting potentials that can make them a potential candidate to be employed as microbial inoculants for protecting plants against phytopathogens, environmental stress and increasing plant growth and productivity.

## Introduction

The rhizosphere is a region in the soil near the plant root, it is an interface between the soil and plant roots (Fasusi et al. 2021). It can be described as a home for plant roots where it is colonized by an enormous number of microbial species such as bacteria which are influenced by the organic compound that are present in the plant root, which is of great economic importance in promoting plant growth (Odelade and Babalola 2019). During plant germination, an interaction occurs between the plant root and the soil around it. This results in the release of organic matter via the soil, which promotes the activities of microorganisms like bacteria in the plant root region, which could be beneficial to enhance plant growth (Gouda et al. 2018). Plant growth-promoting rhizobacteria (PGPR) are beneficial bacteria inhabiting the rhizosphere of plants (Prasad et al. 2019). The plant rhizosphere consists of numerous PGPR species such as *Bacillus Azospirillum*, *Klebsiella,* and *Paenibacillus azotofixans*., *Pseudomonas*, *Rhizobium*, *Azotobacter Acinetobacter,* and *Bacillus,* which are recognized for their potential to stimulate plant growth (Goswami et al. 2016). These PGPR species possess plant growth-promoting potentials that stimulate native plant growth, yield, and defense through different mechanisms such as phytohormones production indole-3-acetic acid (IAA) production (Asari et al. 2017), Phosphate solubilization (Majeed et al. 2015), production of hydrogen cyanide to suppress the deleterious effect of plant pathogens, production of metabolites, production of siderophore which promotes the uptake of iron and nutrient from the environment and making it available for plant growth through the root system, production of lytic enzyme and antimicrobial compounds (Kejela et al. 2016) or production of the fungicidal compound, competitive with plant pathogens and enhancing plant tolerance to biotic and abiotic stress like metal, temperature, water, and salinity (Tewari and Arora 2013; Zolla et al. 2013). The mechanisms of promoting plant growth and defense by these rhizobacteria could be direct or indirect.

Globally, with an increase in the human population, challenges of food security, soil infertility, and the pressure of increasing food production are emerging (Raklami et al. 2019). Cereals and legumes such as soybean and maize are regarded as food crops of great importance and value in human diet. Their production is reduced due to soil infertility; hence, the demand for food by the increasing world population is not entirely met (Raklami et al. 2019). Therefore, the application of agrochemicals in the modern agricultural system to supply the nutrients needed for plant growth and protection of plants against pathogen attacks worldwide arises. However, the cost of purchase and the harmful effects of these chemicals on the environment is of great concern in agricultural practices (Yadav et al. 2018). Therefore, there is an urgent need to research beneficial soil bacteria as alternative means of increasing plant growth and health, intensifying soil fertility, and protecting plants from pathogenic infections that will ensure environmental safety and provide a long-time balance to the ecosystem (Chauhan et al. 2016).

Interest in the PGPR associated with food crops has dramatically increased. It is considered an alternative in promoting plant growth in different areas such as biofertilizers, biopesticides, and probiotics under favorable and unfavorable conditions. The application of PGPR in ensuring agricultural sustainability is a practice that is cost-effective, environmentally friendly, productive, and more efficient over the use of agrochemicals (Itelima et al. 2018). However, little is known on the functional genes and secondary metabolites that are responsible for plant growth promotion. Consequently, knowledge of PGPR, specific to food crops such as soybeans and maize, their characterization, and plant growth potential in promoting plant growth toward ensuring sustainable agricultural practices is needed.

In light of the detrimental effect of chemical fertilizer application on human health and the environment, this study aimed to isolate and characterize rhizobacterial with multiple plant growth-promoting activity from soybeans and maize rhizosphere, evaluate their response to environmental stress, and antagonistic activities against *Fusarium graminearum* a pathogenic fungus that infects cereals. The ability of the rhizobacteria to enhance maize and soybean in vitro was evaluated and the whole genome sequencing of the best isolates was performed to determine the functional genes and secondary metabolites responsible for plant growth promotion in the bacteria genome. Thus, this study can be a basis for application of rhizobacterial strains for biofertilizers production to enhance plant growth and in ensuring agricultural sustainability.

## Materials and methods

### Study site and collection of soil samples

Soil samples were collected from maize and soybean rhizosphere planted in a cultivated field at North-West University Agricultural-Farm, Molelwane, North-West Province, South Africa. The geographical coordinate of the soil sampling sites is (25° 47′ 25.24056″ S 25° 37′ 8.17464″ E) and (25° 47′ 30.14056″ S 25° 37′ 9.27464″ E). The intact root system of the plants was dug out and the rhizospheric soil that attaches to it was collected in sterile plastic bags using a sterile technique method, labelled, and transported to the Microbial Biotechnology Research Laboratory, North-West, University South Africa in the icebox. The samples were stored at − 20 °C for further analysis.

### Isolation of rhizobacteria from the rhizospheric soil

Isolation of rhizospheric bacteria from maize (*Zea mays* L.) and soybeans (*Glycine max*) rhizosphere was carried out using the agar plate method (Karnwal and Kumar 2012). Approximately 10 g of the soil sample from the rhizosphere of maize was collected aseptically and transferred to 90 ml of sterile distilled water inside a 250 ml conical flask with proper stirring. Exactly, 1 ml from the stock was used for a serial dilution with seven test tubes filled with 9 ml of sterile distilled water and 0.1 ml of the suspension in the test tubes was transferred from suitable dilution on Luria Bertani agar (Sigma-Aldrich, Saint Louis, MO, USA) plate in triplicate and incubated at 28 ± 2 °C for 24 h until colonies developed. After incubation, distinct colonies were picked with a sterile wire loop and streaked onto a pre-sterilized Luria Bertani agar plate, incubated at 28 ± 2 °C for 24 h for purification of the bacterial isolates.

### Morphological and biochemical characterization of rhizobacterial isolates

The isolated rhizobacterial morphological features were examined on Luria Bertani agar plates using pure cultures of the bacterial isolates which were incubated at 28 ± 2 °C for 24 h. The morphology of the colonies like the size, shape, colour, and pigmentation was recorded after incubation. Gram reaction for the bacterial isolates was conducted. The biochemical characterization of the bacterial isolates was conducted this includes catalase test, oxidase test, starch hydrolysis, citrate, nitrate reduction test, Voges Proskauer test, casein test, indole test, using the standard procedure described by (Clarke and Cowan 1952). The utilization of carbohydrates by the bacterial isolates was conducted using different carbon sources such as maltose, lactose, glucose, sucrose, fructose, xylose, and galactose.

### In vitro plant growth-promoting traits test

#### Production of indole-3-acetic acid (auxin activity test)

Indole-3-acetic acid production by the rhizobacterial species was determined using the method described by Gordon and Weber (1951) with little modifications. Precisely 0.05 L Luria Bertani broth enriched with 0.1% (D)l-tryptophan inside test tubes was inoculated with a freshly grown overnight culture of each bacterial isolate (500 µl) incubated using a shaking incubator at 30 °C and 180 rpm for 48 h in the dark. After incubation, the bacterial culture was centrifuged at 1000 rpm using a cold centrifuge at 4 °C for 10 min. After centrifugation, 2–3 drops of orthophosphoric acid and 4000 µl of Salkowski reagent (0.05 L of 35% perchloric acid and 1 ml of 0.5 M FeCl_3_ · 6H_2_O) was added to 2000 µl of the supernatant (Ahmad et al. 2008). The mixture was incubated in the dark for 30 min and observed for pink coloration which is an indication of IAA production.

#### Determination of phosphate solubilization

Phosphate solubilization by the bacterial isolates was evaluated according to the method described by Verma et al. (2001). Pikovskaya agar compounded consisting of the following composition/litres: (KCl 200 mg, MgSO_4._7H_2_O 100 mg, MnSO_4_.H_2_O 500 mg, Ca_3_(PO_4_)_2_ 5 g, FeSO_4_.7H_2_O 1 mg, yeast extract 500 mg, glucose 10 g, agar 15 g, (NH_4_)_2_SO_4_ 0.5 g) the pH of the mixture was adjusted to pH 7 using digital pH meter and sterilized at 121 °C for 15 min. Exactly 20 ml of the sterilized medium was poured inside the Petri dishes and left for a while to solidify. Pure culture of the bacterial colony was inoculated on the Pikovskaya agar using the spot inoculation method and Petri plates were incubated at 30 °C for 5–7 days. The observation of clear zone around the bacterial colony was an indication of positive results.

#### Hydrogen cyanide production

Production of hydrogen cyanide (HCN) by the bacterial isolates was conducted using the method described by Castric (1975). Each bacterial isolate was inoculated on sterile LB agar supplemented with glycine (4.4 g/L) using the streaking method. Whatman filter papers were sterilized and dipped inside an alkaline picric acid solution (2% Na_2_CO_3_ in 0.5% picric acid). The filter paper containing the picric acid solution was placed on the upper lid of the Petri plates containing the bacterial inoculum. Before incubation, the Petri plates were sealed with parafilm and incubated at 30 ± 1 °C for 96 h. After incubation, the filter paper was examined for colour change from yellow to red-brown, which was an indication for hydrogen cyanide production.

#### Ammonia production

The bacterial isolates were examined for ammonia production using the method described by Cappuccino and Sherman (1992). Evaluation for ammonia production was conducted by inoculating an overnight culture of each bacterial isolates in 10 ml of sterile peptone water prepared according to the manufacturer's instruction and were incubated at 30 °C for 48 h on a shaking incubator. After incubation, 0.5 ml Nessler’s reagent with the following composition (H_2_O 79.65%, KI 3.49%, NaOH 12.49%, HgI_2_ 4.37%) was added to the samples. The development of slight yellow to brownish colour was an indicator of ammonia production.

#### Production of siderophore

The production of siderophore by the bacterial isolates was determined using chrome-azurol S (CAS) as an indicator dye following the method of Schwyn and Neilands (1987). Briefly, the sterile CAS agar plate was prepared with the following composition (60.5 mg chrome-azurol S dye in 50 ml H_2_O) and mixed with 10 ml iron (III) solution prepared as follows (1 Mm FeCl_3_.6H_2_O in 10 mM HCl). The solution mixture was gently added to hexadecyltrimethylammonium bromide (HDTMA) (72.9 mg in 40 ml H_2_O) and sterilized at 121 °C for 15 min. Approximately100 ml of CAS dye solution was poured into 900 ml of LB agar. Before sterilization of the LB agar, the pH was adjusted to 6.8 using a digital pH meter, sterilized at 121 °C for 15 min, the sterile CAS agar medium was poured into the Petri plate and allowed to solidify. After solidification, the CAS agar plate was spot inoculated with a 24 h old bacteria colony and incubated at 30 °C for 48–72 h. After incubation, the plate was observed for colour change in the medium from blue to an orange or yellow zone of clearance around the bacterial colonies, which was an indication for the production of siderophore.

#### Exopolysaccharide production

The production of exopolysaccharides by the bacterial isolates was evaluated following the method described by Paulo et al. (2012) with few modifications. LB agar was prepared according to the manufacturer's instruction and was amended with 10% sucrose. The pH of the mixture was adjusted to 7 using a digital pH meter before sterilization. The medium was sterilized at 121 °C for 15 min using an autoclave. Sterile Whatman filter paper was cut into 6 mm in diameter and was placed on solidified nutrient agar amended with 10% sucrose in Petri dishes. After that, 2 µl of 24 h old bacterial culture was inoculated on the sterile filter paper placed on agar inside the Petri plate, and the plates were incubated at 30 °C for 2 days. After incubation, plates were observed for the formation of mucoid colonies around the filter paper, which was an indication of the production of exopolysaccharide.

#### Evaluation of antifungal activity of the bacteria isolates

The bacterial isolates were evaluated for antagonistic activities against pathogenic fungi belonging to the genera of *Fusarium* using the dual culture techniques described by Skidmore and Dickinson (1976). *Fusarium graminearum* was collected from the Microbial Biotechnology Laboratory, North-West University, South Africa. The fungus was grown on potato dextrose agar (PDA) plates and incubated at 30 °C for 7 days to ensure that the mycelium of the fungus fully grew on the plate. The fungus was picked using a sterile cock borer and syringe and placed at the centre of a Petri plate containing solidified sterile PDA. Simultaneously, an overnight grown culture of the bacterial isolate was inoculated by streaking method at 2.5 cm from the center of the Petri plate. The control plate was maintained by placing the pathogenic fungus on the PDA agar plate without bacteria. The plates were incubated at 30 °C in an incubator for 7 days and were observed for inhibition zone. The percentage of the fungal mycelial inhibition by the bacterial was calculated using the formula described by Noumavo et al. (2015):

$$\mathrm{Percentage\,growth\,inhibition }\left(\%\right)=\frac{V1-V2}{V1}\times 100$$

where V1 = The diameter of the fungus growth (control) and V2 = the diameter of the fungus growth in the dual culture plate.

#### Genomic sequencing of the bacterial isolates using 16S rRNA analysis

The genomic DNA from each bacterial isolate was extracted using the Zymo soil microbe’s extraction kit (Zymo Research, USA) following the manufacturers instruction.

PCR gene amplification was conducted using a reaction volume of 25 µl containing (0.5 µl forward primer, 0.5 µl reverse primer, 12.5 µl PCR master mix, 2 µl DNA template, 9.5 µl nuclease-free water). Amplification was conducted using 16S rRNA universal primer for bacteria (341F) (5′-CCTACGGGAGGCAGCAG-3′), and (907 R) (5′-CCCCGTCAATTCCTTTGAGTTT-3′) (Fukuda et al. 2016). PCR was performed using the Bio-Rad C 1000 touch thermal cycler and it was programmed at 94 °C for 2 min for initial denaturation, then 94 °C for 30 s, annealing temperature at 59 °C for 1 min, extension at 72 °C in 2 min for 35 cycles, and a final extension at 72 °C in 8 min.

A 1.5% agarose gel containing ethidium bromide was prepared and used to check the quality of the PCR products. The gel was observed using a gel documentation system (Gel Doc 2000, Bio-Rad, USA). Thereafter, PCR products were analyzed for sequencing with the specific universal 16S rRNA primers (341 F) (5′-CCTACGGGAGGCAGCAG-3′), and (907 R) (5′-CCCCGTCAATTCCTTTGAGTTT-3′) at Inqaba Biotechnology Laboratory, South Africa. The sequence from each region of each bacterial isolates was cleaned using Chromas Lite Version 2.1 and edited with Bio Edit Sequence Alignment Editor (Aremu and Babalola 2015; Hall 1999). The consensus sequence was generated and Blast on the NCBI database (www.ncbi.nlm.nih.gov/Blast.cgi) using the Basic Alignment Search Tool (Blastn) for identification of the closest representative strains of the bacterial isolates. The result of the sequence obtained was compared with other sequences in the NCBI GenBank data. The consensus sequence of the bacterial isolates was deposited in the NCBI GenBank database.

#### Effect of the rhizobacterial strains on in vitro seed germination

The effect of the rhizobacteria strains on in vitro seed germination of maize and soybean seed was evaluated using the method described by Gholami et al. (2009) with little modification. Petri dishes were prepared by placing sterile filter paper inside them. Thereafter, the Petri dishes were inoculated with 10 ml of each rhizobacterial suspension (10^7^ CFU/ml) in replicates and were inoculated with 10 sterile maize (Capstone seed, Nelson Choice variety) and soybean (Pannar seed., PAN 1532 variety) seeds as described by Madhaiyan et al. (2007) while the control plate was inoculated with sterile distilled water. The seeds were incubated at 28 °C for 7 days in a growth chamber. After incubation, the percentage seed germination, of the maize and soybean seedlings were measured using the formula below:

$${\rm Percentage\,seed\,germination} (\%)=\frac{GS}{Z}\times 100$$

where GS = Number of seeds that germinated after 7 days and.

Z = The total number of seeds in each Petri dish.

#### Bacterial tolerance to different pH

Tolerance of bacterial isolates to different pH was evaluated by preparing LB broth according to the manufacturer’s instruction, the pH of the LB broth was adjusted to acidic, neutral, and alkaline (4,7,10) using 1MHCl or 1 M NaOH and sterilized at 121 °C for 15 min. Exactly 20 µl from an overnight culture of each bacterial isolate was inoculated into sterile 10 ml LB broth and homogenized. Inoculation of the bacterial isolate was done in triplicate, and the inoculated LB broth was incubated at 30 °C on a rotary shaker at 150 rpm for 48 h. The optical density of the bacterial growth was taken at 600 nm using a UV Spectrophotometer (Thermo Spectronic; Merck).

#### Tolerance of bacterial isolates to different temperatures

The bacterial isolates' tolerance to different temperatures was evaluated by preparing LB broth and sterilized at 121 °C for 15 min. Approximately 5 µl of each bacterial strain was inoculated into 25 ml LB broth and gently vortexed. Inoculation of each bacterial isolate was replicated in triplicate and the inoculated LB broth was incubated at 30, 35, 40 °C. At 24 h of incubation optical density of the bacterial growth was taken at 600 nm using a spectrophotometer (Thermo Spectronic; Meck, South Africa) (Ndeddy Aka and Babalola 2016).

#### Bacterial growth response to different salt concentrations

The effect of bacterial strain tolerance to different salt concentrations (NaCl) was evaluated by preparing LB broth according to the manufacturer's instruction and supplemented with different salt concentrations (1%, 3%, and 5%). The tubes containing 10 ml of the LB broth supplemented with different salt concentrations were inoculated with 100 µl overnight culture of bacterial and were incubated at 30 °C on a shaking incubator at 150 rpm for 72 h. The absorbance of the bacterial growth was measured at 600 nm using a UV Spectrophotometer (Thermo Spectronic; Merck).

#### Bacterial tolerance to different heavy metals

The tolerance of each bacterial isolate to different heavy metals was evaluated in test tubes by preparing the LB broth and supplemented with 100 mg/l of different heavy metals (PbCl_2_, K_2_Cr_2_O_7,_ and CdSO_4_. 8H_2_O). Exactly 10 ml of LB broth containing different heavy metals was dispensed inside test tubes, sterilized at 121 °C for 15 min, inoculated with 1 ml overnight culture of the bacterial strain, and incubated at 30 °C on a rotary shaker at 150 rpm for 72 h. The bacterial growth's optical density was taken after incubation at 600 nm using a UV Spectrophotometer (Thermo Spectronic; Merck) (Ndeddy Aka and Babalola 2016).

### Whole-genome sequencing of rhizobacterial strains

#### Extraction of deoxyribonucleic acid (DNA), sequencing and reads assembly

Based on the result of the in vitro plant growth-promoting test and seed germination potentials of the rhizobacterial strains. Three rhizobacterial strains (*Bacillus* sp. strain OA1, *Pseudomonas rhizosphaerae* strain OA2 and *Pseudomonas* sp. strain OA3) were selected for whole-genome sequencing to determine the functional genes and secondary metabolites that are responsible for plant growth promotion in these rhizobacterial genomes. Fresh culture of the rhizobacterial strains was prepared by streaking the strains separately on sterile LB agar plates incubated at 28 °C for 24 h. Thereafter, the DNA from the fresh bacterial isolates was extracted using Zymo microbe’s extraction kit following the manufacturer's instruction. The extracted DNA was checked for purity by gel electrophoresis and the concentration of the DNA was also checked using the NanoDrop spectrophotometer. Approximately, 4 µl of the extracted DNA from each bacterial isolate was sent for sequencing in an ice pack at Agricultural Research Council (ARC) Biotechnology Platform Pretoria, South Africa for MiSeq system (illumina) sequencing.

Prior, to the DNA sequencing the library was prepared using Illumina Nextera DNA Flex library preparation kit following the manufacturer’s instruction and sequencing was conducted using the MiSeq illumina system. The sequenced read generated from each DNA was in 2 × 250 bp and was visualized using KBase (Arkin et al. 2018). The quality of the reads was checked using Fast QC version 1.0.4 (Andrews 2010), Trimmomatic version 1.2.14 (Bolger et al. 2014) was used to trim low-quality reads and adapter, and the high-quality reads were assembled using SPAdes version 1.2.4 (Nurk et al. 2013). Default parameters were used for all the software unless otherwise stated.

#### Annotation

The genome of *Bacillus* sp. strain OA1, *Pseudomonas rhizosphaerae* strain OA2, and *Pseudomonas* sp. strain OA3 were annotated using Rapid Annotation using Subsystem Technology (RAST) (Overbeek et al. 2014). The RAST permits the annotation of functional genes (manual curation of gene annotation) that are present in the bacterial genome. The result of the annotation was used to determine the contigs location where the genes responsible for plant growth-promoting potentials are located in each rhizobacteria genome. Thereafter, antiSMASH was used to determine the secondary metabolites that are responsible for the plant growth promotion in the bacterial genome. All the data generated have been deposited in the National Center for Biotechnology Information (NCBI) database. The raw reads are available under the Bioproject accession numbers (PRJNA683742- *Bacillus* sp. OA1, PRJNA683763- *Pseudomonas rhizosphaerae* OA2 and PRJNA683768- *Pseudomonas* sp. OA3) and Biosample numbers (SAMN17036205- *Bacillus* sp. OA1, SAMN17036358- *Pseudomonas rhizosphaerea* OA2 and SAMN17036469- *Pseudomonas* sp. OA3). The sequence data obtained in this work have been deposited in the NCBI Sequence Read Archive under accession number (SRX9654894- *Bacillus* sp. OA1, SRX9654938- *Pseudomonas rhizosphaerae* OA2 and SRX9654998- *Pseudomonas* sp. OA3).

### Statistical analysis

All the data obtained from experiments were conducted in triplicates. Data were imputed using Microsoft excel and analysis of variance (ANOVA) was used to analyze the difference among the other parameters and Duncan multiple tests at 5% (*P* < 0.05) probability level was used to check the significance level using SPSS statistical package (v 20.0).

## Result

### Isolation, biochemical and molecular characterization of rhizobacteria

**Fig. 1:**
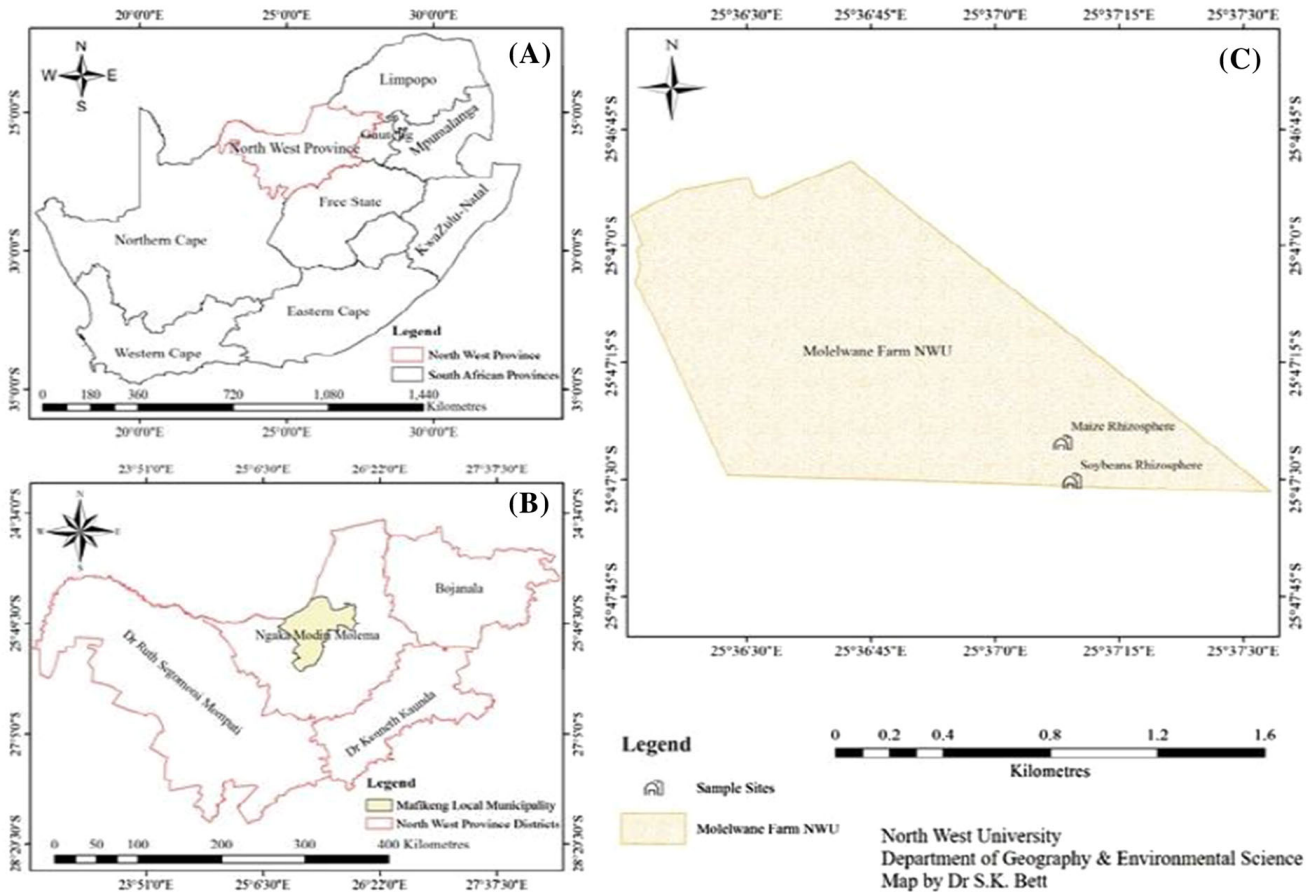
Geographical location of maize and soybeans rhizosphere soil used for isolation of rhizobacterial. **a **The map of South Africa showing North West Province (red sketch) **b** map of North West Province showing the map of Ngaka Modiri Molema district where Mafikeng Local Municipal is located (light yellow region) **c** map of the North-West University Molelwane where maize and soybeans rhizospheric soil sample was collected for rhizobacteria isolation. (Color figure online)

As indicated in Fig. 1, the map showed the geographical location where the rhizospheric soil sample was collected. In this study, twenty-six plant growth-promoting rhizobacteria isolated from maize and soybeans plant rhizosphere were evaluated for their morphological, biochemical characteristics, and plant growth-promoting traits. The bacterial isolates possessed multiple plant growth-promoting characteristics as revealed by their in vitro plant growth-promoting characteristics test conducted. The cultural and biochemical characteristics of the twenty-six bacterial species isolated revealed that the strains were *Bacillus* spp. (21)*., Rhodocyclaceae* bacterium (1), *Enterococcus* spp. (1)***,**** Massilia* spp. (Oxalobacteriaceae) (1), and *Pseudomonas* spp. (2). The number in bracket represent the number of the bacterial species**.** Twenty-two strains were found to be gram-positive MNWU1, SNWU1, MNWU2, MNWU3, SNWU3, SNWU4 MNWU4, MNWU5, SNWU5, MNWU6, MNWU7, SNWU7, SNWU8, SNWU9, MNWU10, SNWU10 SNWU14, MNWU3, MNWU14, MNWU16, MNWU15, SNWU15, except MNWU4, SNWU13, MNWU16, SNWU17 which are gram-negative. All the twenty-six strains were able to show different morphological and biochemical characteristics (Table 1).

**Table 1:** Biochemical characteristics of selected plant growth-promoting rhizobacteria isolates

Isolate code	Cellular shape	Colonial elevation	Colonial edge	Colonial opacity	Colonial surface	Colonial pigmentation	Cellular arrangement
MNWU14	Rod	Raised	Lobate	Translucent	Dull	Whitish Cream	Clusters
SNWU3	Rod	Raised	lobate	Opaque	Dull	Whitish Cream	Clusters
SNWU14	Rod	Raised	Lobate	Translucent	Dull	Whitish Cream	Chain
SNWU15	Rod	Raised	Entire	Opaque	Dull	Whitish Cream	Clusters
SNWU5	Rod	Raised	Lobate	Opaque	Dull	Whitish Cream	Clusters
SNWU13	Rod	Raised	Lobate	Translucent	Smooth	Whitish Cream	Clusters
MNWU5	Rod	Flat	Entire	Opaque	Dull	Whitish Cream	Chain
MNWU16	Rod	Raised	Lobate	Opaque	Pigmented	Whitish Cream	Clusters
SNWU1	Rod	Raised	Entire	Opaque	Smooth	Whitish Cream	Clusters
MNWU4	Rod	Raised	Lobate	Opaque	Smooth	Whitish Cream	Clusters
MNWU15	Rod	Raised	Entire	Opaque	Smooth	Whitish Cream	Clusters
SNWU9	Rod	Raised	Entire	Translucent	Dull	Whitish Cream	Clusters
MNWU2	Rod	Raised	Entire	Translucent	Dull	Whitish Cream	Clusters
MNWU3	Cocci	Flat	Entire	Opaque	Dull	Whitish	Cluster
MNWU6	Rod	Raised	Entire	Opaque	Dull	Creamy White	Clusters
SNWU7	Rod	Raised	Lobate	Translucent	Smooth	Creamy White	Clusters
MNWU1	Rod	Raised	Lobate	Opaque	Dull	Cream	Custer
SNWU8	Rod	Raised	Lobate	Opaque	Dull	Whitish	Clusters
MNWU10	Rod	Flat	Lobate	Opaque	Dull	Whitish	Clusters
SNWU16	Rod	Raised	Lobate	Opaque	Smooth	Creamy White	Clusters
MNWU4	Rod	Raised	Lobate	Opaque	Dull	White	Clusters
MNWU7	Rod	Raised	Entire	Opaque	Smooth	Creamy White	Clusters
MNWU13	Rod	Raised	Entire	Opaque	Smooth	Creamy White	Clusters
SNWU4	Rod	Raised	Entire	Opaque	Smooth	Creamy White	Clusters
SNWU10	Cocci	Raised	Entire	Opaque	Smooth	Creamy White	Clusters
SNWU17	Rod	Raised	Lobate	Opaque	Pigmented	Grey	Clusters

Gram’s staining	Motility test	Spore staining	Catalase	Starch Hydrolysis	Citrate	VP	Casein	Indole
+	+	+	+	+	+	+	+	−
+	+	+	+	+	+	+	+	−
+	+	+	+	−	+	+	+	−
+	+	+	+	+	+	−	−	−
+	+	+	+	+	+	−	+	−
−	−	−	+	+	−	+	−	−
+	+	+	+	+	−	−	+	−
−	−	−	+	−	+	−	−	−
+	−	−	+	+	+	−	+	−
−	+	−	+	+	+	−	+	−
+	+	+	+	−	−	−	−	−
+	+	+	+	+	+	−	+	−
+	+	+	+	+	−	−	+	−
+	−	−	+	−	+	−	+	−
+	+	+	+	+	−	−	+	−
+	−	+	+	+	−	−	−	−
+	+	+	+	+	−	−	+	−
+	+	+	+	+	+	−	+	−
+	+	+	+	+	+	−	+	−
+	+	+	+	−	−	−	−	−
+	+	+	+	−	+	−	+	−
+	−	−	+	+	+	−	+	−
+	+	+	+	−	+	−	+	−
+	+	+	+	+	+	−	+	−
+	+	+	+	+	+	−	+	−
−	−	−	+	−	+	−	+	−

Isolate code	Nitrate reduction	Oxidase	Carbohydrates Utilization				Fructose	Xylose	Galactose
			Maltose	Lactose	Glucose	Sucrose			
MNWU14	+	+	+	+	+	+	+	−	−
SNWU3	+	−	+	−	+	−	−	−	−
SNWU14	+	+	+	−	+	+	−	−	−
SNWU15	+	+	+	−	+	−	+	−	−
SNWU5	+	+	+	+	+	+	+	−	−
SNWU13	+	−	+	−	+	+	−	−	−
MNWU5	+	+	+	−	+	−	−	−	−
MNWU16	+	+	+	−	−	+	−	−	−
SNWU1	+	−	+	+	+	+	+	−	−
MNWU4	+	+	+	−	+	−	+	−	+
MNWU15	+	−	+	−	+	−	−	−	−
SNWU9	+	+	+	+	+	+	+	−	−
MNWU2	+	+	+	−	+	+	+	−	−
MNWU3	+	+	+	+	+	+	+	−	−
MNWU6	+	+	+	−	+	+	+	−	−
SNWU7	+	−	+	+	+	+	+	−	−
MNWU1	+	+	+	−	+	+	+	+	−
SNWU8	+	−	+	−	+	+	−	−	−
MNWU10	+	−	+	+	+	+	+	−	−
SNWU16	+	+	+	−	+	+	−	−	−
MNWU4	+	+	+	+	+	+	+	−	+
MNWU7	+	−	+	+	+	−	+	−	−
MNWU13	−	+	+	−	+	−	+	−	−
SNWU4	+	+	+	−	+	+	+	−	−
SNWU10	+	+	+	−	+	+	+	−	−
SNWU17	+	+	+	−	+	+	+	−	−

− = Negative, +  = Positive

### In vitro plant growth-promoting activities of the bacterial isolates

Rhizobacteria promote plant growth by different mechanisms, which include phosphate solubilization, ammonia production, production of hydrogen cyanide production of exopolysaccharide, production of siderophore that make iron unavailable to plant pathogens and enhancing the secretion of plant hormones like indole-3- acetic acid. Among the bacterial species isolated from maize and soybean rhizosphere, 46% showed a significant zone of clearance on Pikovskaya agar used in the identification of phosphate solubilization. The result of the IAA production revealed that all the rhizobacterial strains were able to produce IAA in a medium amended with L-tryptophan. Moreover, all the bacterial strains showed a positive reaction for ammonia and siderophore test using peptone water. In this study, the bacterial isolates were all positive to the exopolysaccharide test responsible for their tolerance to different environmental stress (Table 2).

**Table 2:** Selected rhizobacterial isolates with multiple plant growth-promoting traits

Isolates code	Phosphate solubilization	HCN production	Siderophore production	Ammonia production	IAA production	Exopolysaccharide production
MNWU14	−	−	+	+	+	+
SNWU3	+	+	+	+	+ +	+
SNWU14	+	−	+	+	+	+
SNWU15	+	+	+ +	+	+	+
SNWU5	+	−	+	+	+ +	+
SNWU13	−	−	+	+	+ +	+
MNWU5	−	+	+	+	+ +	+
MNWU16	+	−	+	+	+ +	+
SNWU1	+	−	+	+	+ +	+
MNWU4	−	+	+	+	+	+
MNWU15	+	−	+ +	+	+	+
SNWU9	+	−	+	+	+	+
MNWU2	−	−	+	+	+ +	+
MNWU3	+	+	+	+	+	+
MNWU6	−	+	+	+	+ +	+
SNWU7	−	−	+	+	+ +	+
MNWU1	−	−	+	+	+	+
SNWU8	−	−	+	+	+ +	+
MNWU10	−	−	+	+	+	+
SNWU16	−	−	+ +	+	+	+
MNWU4	+	−	+	+	+ +	+
MNWU7	−	−	+	+	+	+
MNWU13	+	+	+	+	+ +	+
SNWU4	−	+	+ +	+	+	+
SNWU10	−	−	+	+	+ +	+
SNWU17	+	+	+	+	+ +	+

+  = presence of trait, +  +  = highly presence of trait, − = absence of trait

### Antifungal activity

**Fig. 2:**
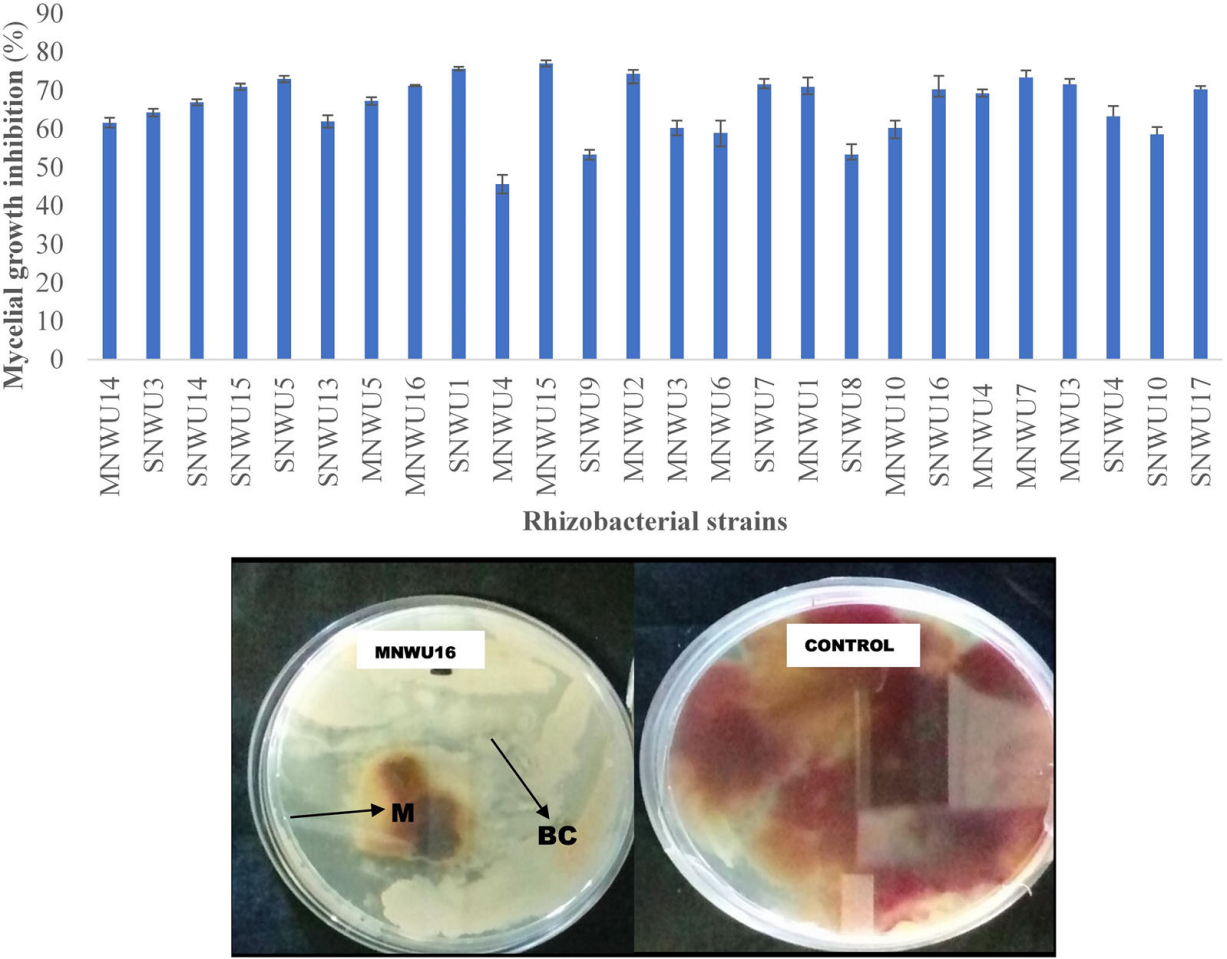
Antifungal activity of the rhizobacterial isolates against *Fusarium graminearium*. M = Fungal mycelia, BC = bacterial colony, and the control plate

The antifungal activity of the bacterial isolates against *Fusarium graminearium,* a fungus pathogen that infects maize using a dual assay culture method, was positive for all the bacterial. Based on the in vitro antifungal activity,46.1% of rhizobacterial strains recorded a percentage of fungal mycelium inhibition of 70% and above while 50.9% of the isolates resulted in the percentage of fungal mycelium inhibition of less than 70% (Fig. 2).

### Molecular characterization of the bacterial isolates using 16S rRNA primer

The identification of the rhizobacterial strains with multiple plant growth-promoting traits characterize with molecularly using 16S rRNA universal primer for bacterial characterization, as shown in (Table 3). The consensus sequence obtained from each isolate were BLAST using Blastn and submitted to NCBI GenBank. Based on 16S rRNA gene characterization, the bacterial species isolated from the rhizosphere of maize and soybean plants were divided into Firmicutes, Beta, and Gammaproteobacteria classes. The bacterial isolates belong to different genera such as *Bacillus*, *Enterococcus*, *Massilia, Pseudomonas,* and *Rhodocyclaceae.* All the sequences generated by the bacteria isolates were deposited in the National Center for Biotechnology Information (NCBI) GenBank database with different accession numbers, in which the *Bacillus* genera have the highest diversity with different species which include *Bacillus simplex* MT982681 (MNWU14)*, Bacillus megaterium* MT982682 (SNWU3), *Bacillus paramycoides* MT982683 (SNWU14), *Bacillus thuringiensis* MT982684 (SNWU15), *Bacillus* sp MT989395 (SNWU5), *Bacillus paramycoides* MT982685 (MNWU5), *Bacillus megaterium* MT982687 (SNWU1), *Bacillus aryabhattai* MT982688 (MNWU15)*, Bacillus cereus* MT982689 (SNWU9), *Bacillus thuringiensis* MT982690 (MNWU2), *Bacillus thuringiensis* MT982691 (SNWU6), *Bacillus subtilis* MT982692 (SNWU7), *Bacillus thuringiensis* MT982693 (MNWU1), *Bacillus cereus* MT982694 (SNWU8), *Bacillus cereus* MT982695 (MNWU10), *Bacillus pumilus* MT982696 (SNWU16)*, Bacillus albus* MT801081 (MNWU4)*, Bacillus paramycoides* MT801080 (MNWU7), *Bacillus siamensis* MT801076 (MNWU3)*, Bacillus atrophaeus* MT801077 (SNWU4),*, Bacillus wiedmannii* MT801078 (SNWU10). Follow by, *Pseudomonas* genera with the diversity of two species, which are *Pseudomonas* sp. MT982686 (MNWU16), and *Pseudomonas rhizosphaerae* MT801079 (SNWU17), while *Rhodocyclaceae* bacterium MT989397 (MNWU4), *Enterococcus* spp. MT989398 (MNWU3), and *Massilia* spp. MT989396 (SNWU13) were represented by one isolate.

**Table 3:** 16S rRNA sequence identification of the species closely associated with bacterial isolate using the Blastn program

S/N	Isolates Code	Source of Isolate	Accession No	Percentage Similarity (%)	E-value	Query cover	Strains	Genus (Closest representative species)
1	MNWU14	Maize	MT982681	98	0.0	98	AO4	*Bacillus simplex*
2	SNWU3	Soybean	MT982682	100	0.0	99	AO5	*Bacillus megaterium*
3	SNWU14	Soybean	MT982683	99.02	0.0	100	AO6	*Bacillus paramycoides*
4	SNWU15	Soybean	MT982684	98	0.0	100	AO7	*Bacillus thuringiensis*
5	SNWU5	Soybean	MT989395	100	0.0	99	AO1	*Bacillus* spp
6	SNWU13	Soybean	MT989396	99	1e−173	87	AO8	*Massilia* spp
7	MNWU5	Maize	MT982685	95.60	0.0	97	AO9	*Bacilluss paramycoides*
8	MNWU16	Maize	MT982686	100	0.0	100	AO3	*Pseudomonas* spp
9	SNWU1	Soybean	MT982687	99.60	0.0	100	AO10	*Bacillus megaterium*
10	MNWU4	Maize	MT989397	99	1e−148	96	AO11	*Rhodocyclaceae bacterium*
11	MNWU15	Maize	MT982688	99.80	0.0	100	AO12	*Bacillus aryabhattai*
12	SNWU9	Soybeans	MT982689	100	0.0	100	AO13	*Bacillus cereus*
13	MNWU2	Maize	MT982690	100	0.0	99	AO14	*Bacillus thuringiensis*
14	MNWU3	Maize	MT989398	100	0.0	97	AO15	*Enterococcus sp*
15	MNWU6	Maize	MT982691	99.80	0.0	100	AO16	*Bacillus thuringiensis*
16	SNWU7	Soybean	MT982692	97.68	0.0	100	AO17	*Bacillus subtilis*
17	MNWU1	Maize	MT982693	99.56	0.0	100	AO18	*Bacillus thuringiensis*
18	SNWU8	Soybean	MT982694	99.26	0.0	100	AO19	*Bacillus cereus*
19	MNWU10	Maize	MT982695	99.81	0.0	100	AO20	*Bacillus cereus*
20	SNWU16	Soybean	MT982696	97.60	0.0	100	AO21	*Bacillus pumilus*
21	MNWU4	Maize	MT801081	100	0.0	100	AO22	*Bacillus albus*
22	MNWU7	Maize	MT801080	97	0.0	98	AO23	*Bacillus paramycoides*
23	MNWU13	Maize	MT801076	100	0.0	100	AO24	*Bacillus siamensis*
24	SNWU4	Soybean	MT801077	100	0.0	100	AO25	*Bacillus atrophaeus*
25	SNWU10	Soybean	MT801078	100	0.0	100	AO26	*Bacillus wiedmannii*
26	SNWU17	Maize	MT801079	99	0.0	97	AO2	*Pseudomonas rhizosphaerae*

### Tolerance to heavy metal, salt, temperature and pH

The bacterial isolate's tolerance to heavy metal was shown in Table 4. Based on the result obtained from this study, the bacterial isolate exhibited different tolerance to the heavy metals used. In this study, the bacterial isolate with an optical density above 0.45 was considered tolerant to heavy metal. The highest percentage in the bacterial tolerance to heavy metal was reported in chromium (61.53%), followed by lead (30.76%), while the lowest percentage tolerance was recorded in cadmium (3.8%), respectively. Cadmium was reported in this study to be toxic to the bacterial isolates. Therefore, the tolerance of the bacterial isolates characterized in this finding reduced as follows:

$${\text{Cr}}\, > \,{\text{Pb}}\, > \,{\text{Cd}}$$

**Table 4:** Tolerance of bacterial isolates to heavy metals

Isolates code	Pb	Cr^2+^	Cd^+^
MNWU14	0.58 ± 0.06^cde^	0.66 ± 0.06^cd^	0.11 ± 0.01^jk^
SNWU3	0.38 ± 0.05^fgh^	0.30 ± 0.05^kl^	0.26 ± 0.03^def^
SNWU14	0.28 ± 0.06^hij^	0.56 ± 0.05^def^	0.15 ± 0.02^hijk^
SNWU15	0.21 ± 0.06^jkl^	0.45 ± 0.05^ghi^	0.1 ± 0.01^ijk^
SNWU5	0.15 ± 0.04^klm^	0.52 ± 0.03^fgh^	0.20 ± 0.04^fghij^
SNWU13	0.04 ± 0.01^m^	0.10 ± 0.01^n^	0.1 ± 0.05^fghij^
MNWU5	0.15 ± 0.02^klm^	0.54 ± 0.02^efg^	0.14 ± 0.02^hijk^
MNWU16	0.32 ± 0.05^hij^	0.43 ± 0.05^hij^	0.26 ± 0.03^defg^
SNWU1	0.68 ± 0.06^bc^	0.58 ± 0.06^def^	0.23 ± 0.01^defgh^
MNWU4	0.73 ± 0.07^b^	0.87 ± 0.07^a^	0.23 ± 0.01^defgh^
MNWU15	0.46 ± 0.03^efg^	0.578 ± 0.03^def^	0.26 ± 0.02^defg^
SNWU9	0.26 ± 0.04^hijkl^	0.36 ± 0.03^ijk^	0.17 ± 0.03^ghijk^
MNWU2	0.35 ± 0.05^ghi^	0.48 ± 0.05^fgh^	0.07 ± 0.01^k^
MNWU3	0.63 ± 0.01^bcd^	0.72 ± 0.01^bc^	0.45 ± 0.02^b^
MNWU6	0.26 ± 0.03^hijkl^	0.42 ± 0.03^hij^	0.12 ± 0.01^ijk^
SNWU7	0.28 ± 0.03^hijk^	0.22 ± 0.02^lm^	0.36 ± 0.02^c^
MNWU1	0.21 ± 0.03^jkl^	0.57 ± 0.03^defg^	0.19 ± 0.05^fghij^
SNWU8	0.22 ± 0.01^ijkl^	0.52 ± 0.01^fgh^	0.16 ± 0.02^hijk^
MNWU10	0.13 ± 0.01^lm^	0.33 ± 0.01^jkl^	0.24 ± 0.01^defgh^
SNWU16	0.50 ± 0.03^def^	0.57 ± 0.03^def^	0.30 ± 0.05^cde^
MNWU4	0.30 ± 0.03^hij^	0.32 ± 0.03^jkl^	0.11 ± 0.01^jk^
MNWU7	0.95 ± 0.02^a^	0.81 ± 0.02^ab^	0.32 ± 0.01^cd^
MNWU13	0.14 ± 0.02^klm^	0.47 ± 0.02^fgh^	0.17 ± 0.01^ghijk^
SNWU4	0.98 ± 0.04^a^	0.71 ± 0.04^bc^	0.22 ± 0.01^efghi^
SNWU10	0.19 ± 0.01^jkl^	0.16 ± 0.01^mn^	0.21 ± 0.01^efghi^
SNWU17	0.49 ± 0.05^ef^	0.62 ± 0.05^cde^	0.67 ± 0.01^a^

Values are expressed as mean ± SE (n = 3), there is no significant difference (*P* > 0.05) with values having the same letter within a column

The bacterial isolate tolerance to different salt concentrations was shown in Table 5. In this study, the tolerance revealed that virtually all the bacterial strains showed high tolerance in a culture medium amended with a salt concentration of 1% and 3%. The percentage of bacterial isolates that recorded increased in their optical density in culture medium amended with 5% salt concentration was 19.23%, while the percentage of the bacterial isolate with decreased in their optical density in medium amended with 5% salt concentration 80.76%.

**Table 5:** Response of bacterial isolates to different salt concentration

Isolates code	1%	3%	5%
MNWU14	1.80 ± 0.06^cd^	1.84 ± 0.05^ab^	1.45 ± 0.05^gh^
SNWU3	1.72 ± 0.05^d^	1.74 ± 0.07^ab^	1.67 ± 0.06^efg^
SNWU14	2.06 ± 0.03^abcd^	1.81 ± 0.06 ^ab^	2.01 ± 0.29^abcd^
SNWU15	2.05 ± 0.03^abcd^	1.77 ± 0.05^ab^	1.92 ± 0.01^abcde^
SNWU5	1.99 ± 0.06^abcd^	1.99 ± 0.51^ab^	1.89 ± 0.04^abcdef^
SNWU13	1.04 ± 0.02^e^	1.50 ± 0.28^ab^	1.36 ± 0.03^hi^
MNWU5	1.99 ± 0.11^abcd^	1.99 ± 0.51 ^ab^	1.78 ± 0.03^cdef^
MNWU16	2.13 ± 0.02^abc^	2.05 ± 0.02^ab^	1.96 ± 0.05^abcd^
SNWU1	1.32 ± 0.16^e^	1.82 ± 0.01^ab^	2.16 ± 0.09^a^
MNWU4	2.24 ± 0.02^ab^	2.00 ± 0.29^ab^	1.75 ± 0.02^def^
MNWU15	1.94 ± 0.02^bcd^	2.01 ± 0.29^ab^	1.79 ± 0.04^bcdef^
SNWU9	1.78 ± 0.04^cd^	1.50 ± 0.22^ab^	1.39 ± 0.04^hi^
MNWU2	1.93 ± 0.05^abcd^	1.97 ± 0.30^ab^	1.61 ± 0.01^fgh^
MNWU3	2.00 ± 0.29^abcd^	1.94 ± 0.02^ab^	1.85 ± 0.02^bcdef^
MNWU6	2.11 ± 0.06^abcd^	2.08 ± 0.04 ^ab^	2.03 ± 0.01^abc^
SNWU7	1.76 ± 0.41^cd^	1.88 ± 0.04^ab^	1.18 ± 0.03^i^
MNWU1	1.87 ± 0.04^bcd^	1.97 ± 0.05^ab^	1.79 ± 0.05^bcdef^
SNWU8	2.36 ± 0.17^a^	1.94 ± 0.06^ab^	1.16 ± 0.03^i^
MNWU10	2.03 ± 0.02^abcd^	2.11 ± 0.05^ab^	2.07 ± 0.03^ab^
SNWU16	2.06 ± 0.03^abcd^	1.61 ± 0.06^ab^	1.60 ± 0.11^fgh^
MNWU4	1.96 ± 0.03^abcd^	1.92 ± 0.05^ab^	1.94 ± 0.02^abcde^
MNWU7	1.99 ± 0.05^abcd^	2.01 ± 0.29^ab^	1.84 ± 0.01^bcdef^
MNWU13	1.76 ± 0.03^cd^	1.83 ± 0.05^ab^	1.91 ± 0.11^abcde^
SNWU4	2.07 ± 0.02^abcd^	2.15 ± 0.02^ab^	1.86 ± 0.05^bcdef^
SNWU10	1.05 ± 0.03^e^	1.42 ± 0.01^b^	1.39 ± 0.17^hi^
SNWU17	2.02 ± 0.01^abcd^	2.17 ± 0.03^a^	2.04 ± 0.02^abc^

Values are expressed as mean ± SE (n = 3), there is no significant difference (*P* > 0.05) with values having the same letter within a column

In this study, the bacterial species isolated were evaluated for their tolerance to a different temperature ranging from 30 to 40 °C as shown in Table 6. The growth of the bacterial isolate to diverse temperature range evaluated in this study was significant (*P* < 0.05). At temperature of 30 °C and 35 °C most of the bacterial exhibited high optical density, which could be regarded as the optimum temperature for their growth. At the temperature of 40 °C, the optical density of the bacterial isolate was reduced, respectively.

**Table 6:** Response of bacterial isolates to different temperature

Isolate code	30 °C	35 °C	40 °C
MNWU14	0.30 ± 0.05^jk^	0.58 ± 0.04^ghijk^	0.12 ± 0.01^hi^
SNWU3	0.42 ± 0.01^hi^	0.31 ± 0.01^m^	0.49 ± 0.05^a^
SNWU14	0.51 ± 0.01^gh^	0.58 ± 0.04^ghijk^	0.33 ± 0.01^de^
SNWU15	0.24 ± 0.02^k^	0.85 ± 0.02^bc^	0.33 ± 0.01^de^
SNWU5	0.59 ± 0.04^k^	0.48 ± 0.04^jkl^	0.29 ± 0.04^def^
SNWU13	0.27 ± 0.01^k^	0.44 ± 0.03^l^	0.09 ± 0.01^i^
MNWU5	0.77 ± 0.03^d^	0.82 ± 0.05^cd^	0.24 ± 0.05^efg^
MNWU16	0.40 ± 0.06^ij^	0.86 ± 0.03^bc^	0.19 ± 0.04^fghi^
SNWU1	0.39 ± 0.03^ij^	0.57 ± 0.03^hijk^	0.25 ± 0.02^efg^
MNWU4	0.95 ± 0.02^bc^	0.53 ± 0.01^ijkl^	0.37 ± 0.03^cdd^
MNWU15	1.03 ± 0.01^b^	0.60 ± 0.05^ghijk^	0.34 ± 0.02^de^
SNWU9	0.57 ± 0.03^fg^	0.87 ± 0.01^bc^	0.38 ± 0.04^bcd^
MNWU2	0.26 ± 0.03^k^	0.42 ± 0.01^lm^	0.19 ± 0.05^fghi^
MNWU3	1.19 ± 0.05^a^	0.67 ± 0.03^fgh^	0.47 ± 0.03^ab^
MNWU6	0.66 ± 0.05^ef^	0.47 ± 0.03^kl^	0.33 ± 0.01^de^
SNWU7	0.21 ± 0.01^k^	0.16 ± 0.02^n^	0.10 ± 0.03^hi^
MNWU1	0.26 ± 0.03^k^	0.59 ± 0.04^ghijk^	0.16 ± 0.02^ghi^
SNWU8	0.46 ± 0.03^hi^	0.58 ± 0.04^ghijk^	0.21 ± 0.01^fgh^
MNWU10	0.29 ± 0.04^jk^	0.96 ± 0.03^a^	0.15 ± 0.02^ghi^
SNWU16	0.46 ± 0.03^hi^	0.71 ± 0.05^def^	0.17 ± 0.03^ghi^
MNWU4	0.72 ± 0.01^de^	0.61 ± 0.06^ghij^	0.15 ± 0.02^ghi^
MNWU7	0.96 ± 0.01^bc^	0.65 ± 0.02^fghi^	0.34 ± 0.02^de^
MNWU13	0.93 ± 0.02^bc^	0.59 ± 0.05^ghijk^	0.45 ± 0.01^abc^
SNWU4	0.92 ± 0.01^c^	0.76 ± 0.03^cde^	0.37 ± 0.03^bcd^
SNWU10	0.25 ± 0.02^k^	0.19 ± 0.04^n^	0.21 ± 0.01^fgh^
SNWU17	1.20 ± 0.01^a^	0.60 ± 0.05^gh^	0.35 ± 0.02jk

Values are expressed as mean ± SE (n = 3), there is no significant difference (*P* > 0.05) with values having the same letter within a column

The tolerance of the bacterial isolate to different pH range (4, 7, and 10) was evaluated in this study, as shown in Table 7. At pH 4, the optical density of the bacterial isolate above 0.4 was considered tolerance to acidic medium. In this case, 46.15% of the bacterial isolate was tolerant to pH 4, 84.61% tolerance to pH 7, and 7.6% tolerance to pH 10. The result could also enhance the classification of the bacterial isolate into acid and alkaline tolerance microorganisms.

**Table 7:** Response of bacteria isolates to different pH

Isolate code	4	7	10
MNWU14	0.13 ± 0.05^gh^	0.63 ± 0.06^cd^	0.08 ± 0.01^j^
SNWU3	0.16 ± 0.03^gh^	0.63 ± 0.01^cd^	0.15 ± 0.02^fghij^
SNWU14	0.39 ± 0.04^de^	0.52 ± 0.02^def^	0.13 ± 0.01^ghij^
SNWU15	0.16 ± 0.03^gh^	0.80 ± 0.06^b^	0.14 ± 0.02^fghij^
SNWU5	0.44 ± 0.02^d^	0.58 ± 0.01^cde^	0.14 ± 0.01 ^fghij^
SNWU13	0.22 ± 0.01^fg^	0.35 ± 0.03^fgh^	0.13 ± 0.01 ^fghij^
MNWU5	0.16 ± 0.03^gh^	0.34 ± 0.01^hi^	0.10 ± 0.01^ij^
MNWU16	0.25 ± 0.02^fg^	0.42 ± 0.05^fgh^	0.23 ± 0.01^defg^
SNWU1	0.63 ± 0.01^b^	0.71 ± 0.05^bc^	0.10 ± 0.01^ij^
MNWU4	0.65 ± 0.02^b^	0.70 ± 0.06^bc^	0.15 ± 0.02^fghij^
MNWU15	0.09 ± 0.01^h^	0.48 ± 0.01^efg^	0.17 ± 0.03^efghij^
SNWU9	0.42 ± 0.06^de^	0.51 ± 0.05^def^	0.30 ± 0.05^cd^
MNWU2	0.68 ± 0.05^b^	0.68 ± 0.04^bc^	0.19 ± 0.04^efghi^
MNWU3	0.45 ± 0.02^d^	0.68 ± 0.04^bc^	0.34 ± 0.02^c^
MNWU6	0.58 ± 0.04^bc^	0.61 ± 0.05^cde^	0.16 ± 0.02^fghij^
SNWU7	0.49 ± 0.04^cd^	0.65 ± 0.05^cd^	0.16 ± 0.02^fghij^
MNWU1	0.16 ± 0.03^gh^	0.72 ± 0.01^bc^	0.23 ± 0.01^def^
SNWU8	0.46 ± 0.03^d^	0.71 ± 0.06^bc^	0.17 ± 0.03 ^efghij^
MNWU10	0.16 ± 0.03^gh^	0.61 ± 0.05^cde^	0.16 ± 0.03 ^efghij^
SNWU16	0.28 ± 0.02^fg^	0.48 ± 0.05^efg^	0.21 ± 0.04^efgh^
MNWU4	0.13 ± 0.01^gh^	0.35 ± 0.02^ghi^	0.12 ± 0.01^hij^
MNWU7	0.30 ± 0.05^ef^	1.06 ± 0.03^a^	0.26 ± 0.04^cde^
MNWU13	0.44 ± 0.02^d^	0.68 ± 0.04^bc^	0.13 ± 0.01^fghij^
SNWU4	0.87 ± 0.01^a^	1.16 ± 0.03^a^	0.73 ± 0.01^a^
SNWU10	0.37 ± 0.03^de^	0.25 ± 0.02^i^	0.09 ± 0.01^ij^
SNWU17	0.60 ± 0.06^b^	1.05 ± 0.02^a^	0.51 ± 0.05^b^

Values are expressed as mean ± SE (n = 3), there is no significant difference (*P* > 0.05) with values having the same letter within a column

### Effect of the rhizobacterial strains on seed germination

**Fig. 3:**
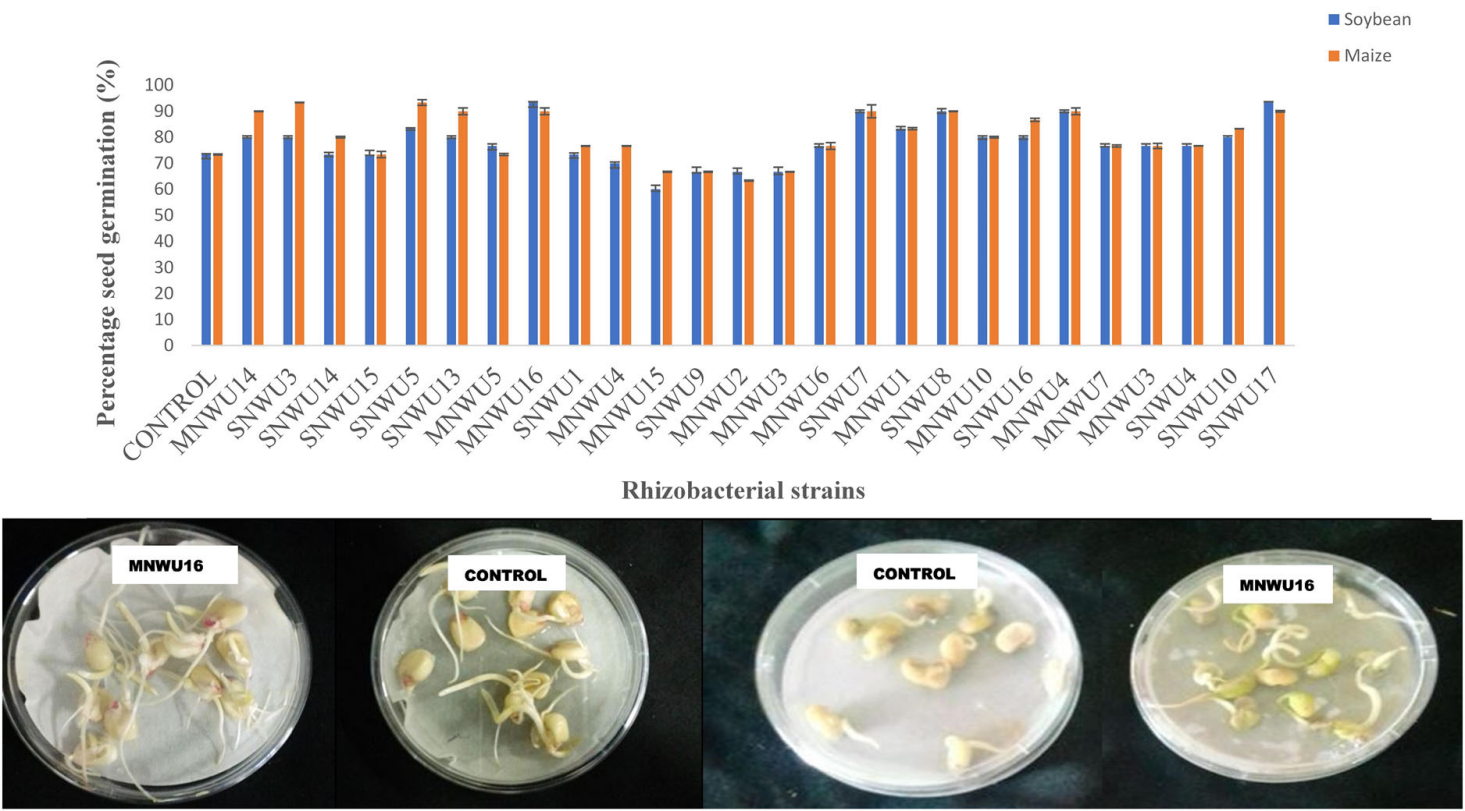
Effect of rhizobacterial strains on in vitro maize and soybeans seed germination

The rhizobacterial strains characterized in this study were able to promote maize and soybean seed under laboratory conditions where this experiment was conducted as shown in Fig. 3**.** The result of the seed germination test revealed the highest seed germination percentage (above 80%) in the *Bacillus* sp. OA1, *Pseudomonas rhizosphaerae* OA2 and *Pseudomonas* sp. OA3. However, some of the bacterial isolates as well as the control showed a lower percentage of germination (below 80%).

### Whole-genome analysis of plant growth-promoting functional genes and secondary metabolite in the rhizobacterial

**Fig. 4:**
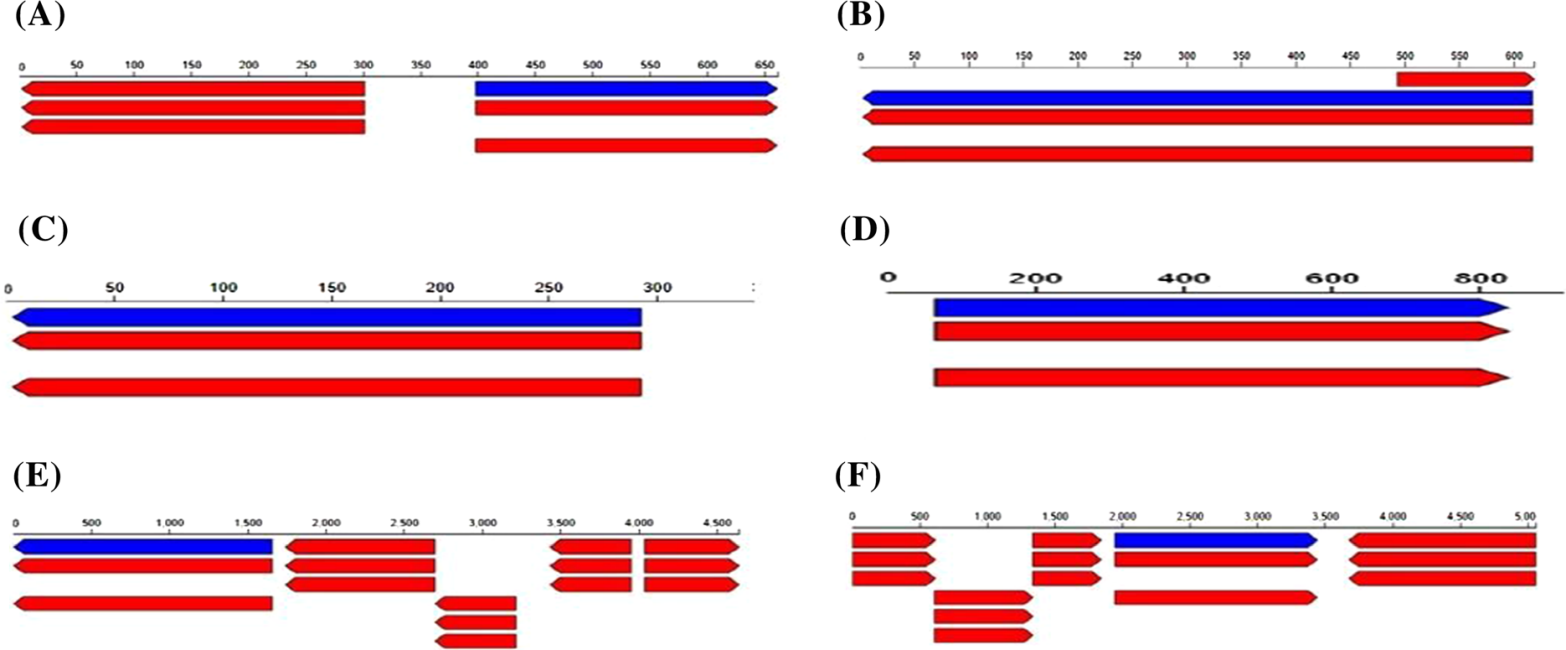
Contigs location of the plant growth promote functional genes in the rhizobacterial genome **a** Phosphoglycolate phosphatase (EC 3.1.3.18) **b** Siderophore biosynthesis non-ribosomal peptide synthetase modules @ Bacillibactin synthetase component F (EC 2.7.7.–) **c** Anthranilate phosphoribosyltransferase (EC 2.4.2.18) **d** Iron siderophore sensor protein **e** Ferrichrome-iron receptor FiuA @ Ferric siderophore receptor, TonB dependent **f** Exopolyphosphatase (EC 3.6.1.11). The blue bars represent the region where the genes are located. (Color figure online)

**Fig. 5:**
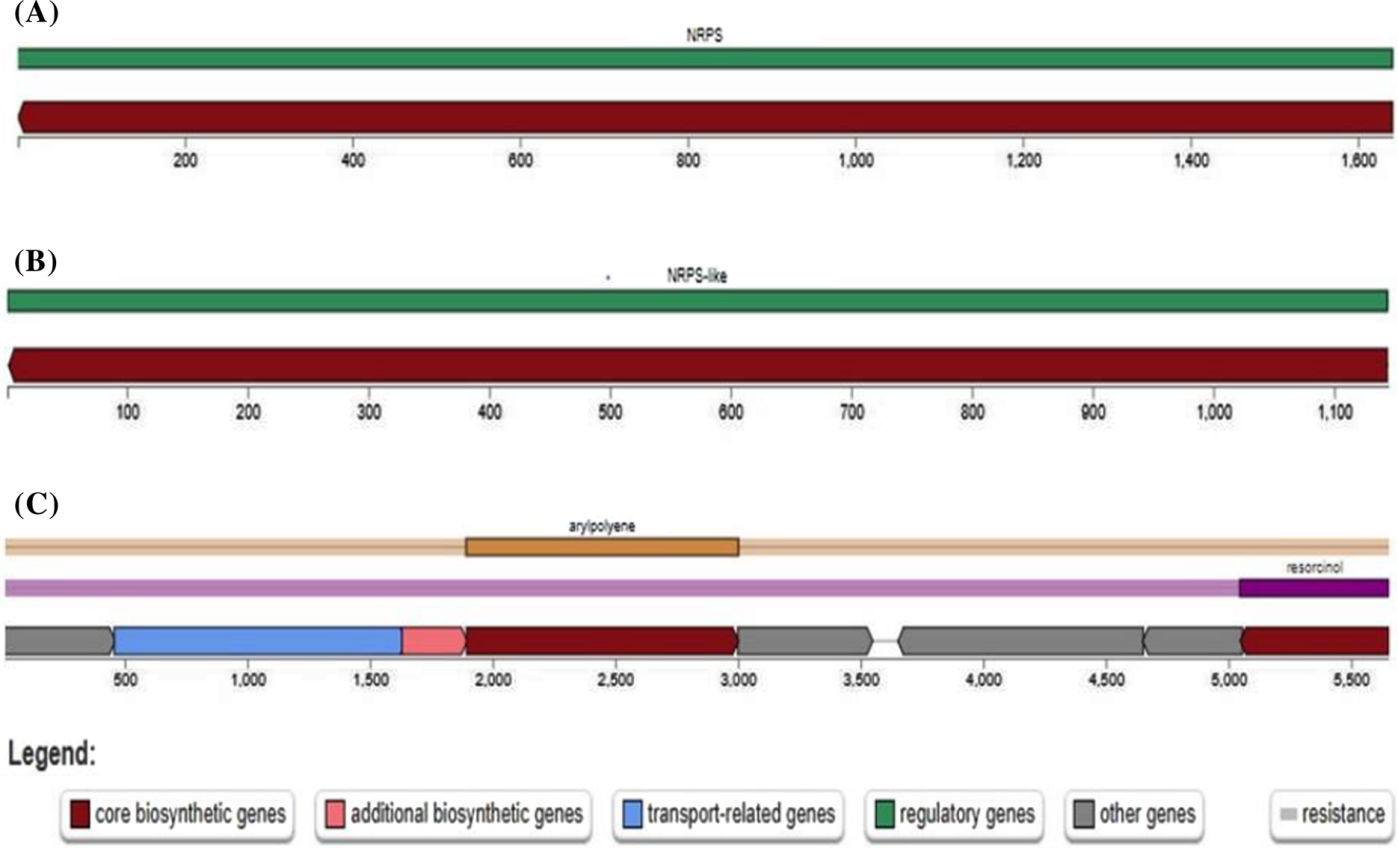
Selected secondary metabolites in the rhizobacterial genome **a** NRPS-*Bacillus* sp. OA1 **b** NRPS-like *Pseudomonas rhizospherea* OA2 **c** Arypolyene, resorcinol- *Pseudomonas* sp. OA3

The functional genes and secondary metabolite that are responsible for plant growth-promoting potential in the rhizobacterial strain's genome as revealed by whole-genome sequencing were reported in Table 8. The region where the plant growth-promoting functional gene and the metabolites are located was reported in Figs. 4 and 5. The blue bar in Fig. 4 represents the contigs location where the plant growth-promoting are located. Among the plant growth-promoting, functional genes selected are genes responsible for phosphate solubilization, siderophore production, and exopolysaccharide production. For the secondary metabolite that is present in the rhizobacterial genome, NRPS, arypolyene, and resorcinol which are crucial in enhancing plant growth promotion were selected.

**Table 8:** Some selected plant growth-promoting functional genes revealed by whole-genome sequencing of the rhizobacterial strains

Rhizobacterial stains	Feature/ ID Type	Function	Ontology	Start	Strand	Protein length	Location
*Bacillus* sp. OA1	CDS.10026/Gene	Phosphoglycolate phosphatase (EC 3.1.3.18)	SSO:000,005,983- Phosphoglycolate phosphatase (EC 3.1.3.18)	398	** + **	264	NODE_2883_length_662_cov_3.704673
	CDS.10284/Gene	Siderophore biosynthesis non-ribosomal peptide synthetase modules @ Bacillibactin synthetase component F (EC 2.7.7.-)	SSO:000,001,137- Bacillibactin synthetase component F (EC 2.7.7.–) SSO:000,007,486- Siderophore biosynthesis non-ribosomal peptide synthetase modules	617	+	615	NODE_3079_length_619_cov_2.752033
*Pseudomonas rhizospherea* OA2	CDS.10018/Gene	Anthranilate phosphoribosyltransferase (EC 2.4.2.18)	SSO:000,000,995- Anthranilate phosphoribosyltransferase (EC 2.4.2.18)	293	−	291	NODE_5433_length_509_cov_1.801047
	CDS.1005/Gene	Iron siderophore sensor protein	SSO:000,004,003- Iron siderophore sensor protein	65	+	777	NODE_239_length_2787_cov_2.212782
*Pseudomonas* sp. OA3	CDS.1624/Gene	Ferrichrome-iron receptor FiuA @ Ferric siderophore receptor, TonB dependent	SSO:000,002,737- Ferric siderophore receptor, TonB dependent	1658	**−**	1656	NODE_229_length_4642_cov_1.975415
	CDS.1404/Gene	Exopolyphosphatase (EC 3.6.1.11)	SSO:000,002,629- Exopolyphosphatase (EC 3.6.1.11)	1944	_**+**_	1503	NODE_187_length_5062_cov_3.014995

## Discussion

The application of agrochemicals in agrarian practices has become deleterious to humans and the environment. Therefore, there is a need to intensify research on new advances in the application of biological and ecologically friendly approaches which involve the use of rhizospheric beneficial bacterial to promote plant growth and improve plant tolerance to environmental stress and phytopathogen (Fasusi and Babalola 2021). The molecular characterization of the bacterial isolates evaluated in this study is crucial for proper identification and description of the bacterial isolates, and it gives vital information about the novelty of the organisms (Adegboye and Babalola 2012). The similarity of the bacterial isolates evaluated in the study met the standard and the specification of the National Center for Biotechnology Information (NCBI), which could enhance the consensus sequence generated from each isolate to be deposited in the NCBI GenBank for allocation of an accession number.

The plant growth-promoting rhizobacterial colonizes plant roots by promoting plant growth and competitively limit the population of harmful soil microorganisms using various mechanisms such as the production of hydrogen cyanide, phosphate solubilization, ammonia production, antibiotics production, siderophore production, and stimulating the production of phytohormones such as IAA (Olanrewaju et al., 2017; Meena et al., 2017).

In this study, it was shown that the rhizosphere of soybeans and maize harbors beneficial soil bacteria with multiple plant growth-promoting features. In previous studies, the presence of rhizospheric bacteria that are of importance in promoting plant growth has been reported (Josephine and Thomas 2019). In this study, the bacterial were screened for in vitro plant growth-promoting characteristics, antifungal potential, and response to environmental stress. The presence of beneficial bacterial in the plant rhizosphere is well established to be crucial for plant growth promotion and defense. For bacterial isolate to be an efficient plant growth-promoting rhizobacteria strain, the bacterial strain must be able to colonize the plant rhizosphere at high population density, which will enable it to exact its beneficial effect on plant development, tolerance to stress, and defense. The isolated and characterized rhizobacterial evaluated in this study were likely to contribute to the promotion of plant growth and enhance plant tolerance to different environmental stress.

On the other hand, rhizobacterial with plant growth-promoting potentials such as bacteria that solubilize phosphate play a crucial in promoting plant growth by converting insoluble phosphorus to soluble phosphatase for plant absorption (Alori et al. 2017). Therefore, soil fertility and plant growth promotion can be enhanced by the application of these phosphate solubilizing microorganisms, and the widespread commercialization of these microorganisms can reduce the cost of purchase of chemical fertilizer, the ill effect associated with its usage, and its application for increasing plant growth. The phosphate solubilization potentials of the rhizobacterial isolates in this study were in agreement with the previous research conducted by Rajkumar et al. (2006) that attributed plant root proliferation and absorption of nutrient such as phosphorus by the plant to the potential of phosphate solubilizing *Bacillus* sp. BA32 and *Pseudomonas* sp. PSA4.

Furthermore, rhizobacteria possessing multiple plant growth-promoting potential can be employed in enhancing plant growth promotion and protecting plant health for an optimum increase in agricultural productivity (Zaidi et al. 2015). The numerous plant growth-promoting ability of *Bacillus* spp. and *Pseudomonas* spp. characterized in the study are consistent with the finding of other researchers (Ahmad et al. 2016; Ndeddy Aka and Babalola 2016). Likewise, the characterization of *Rhodocyclaceae* bacterium with plant growth-promoting potentials from soybeans rhizosphere in this study has been reported by Liu et al. (2019) as rhizosphere soil microbiomes associated with soybeans plant which could contribute to plant growth enhancement. In this study, the production of plant growth-promoting trait by *Enterococcus* spp. agreed with the study of (Anzuay et al. 2017; Mussa et al. 2018) that reported the isolation of *Enterococcus* spp growth-promoting potential and antifungal activity against pathogenic fungi from the rhizosphere of grass pea. *Massilia* spp**.** (Oxalobacteriaceae) characterization from plant rhizosphere in this study for plant growth-promoting activity corroborates with the finding of Soares et al. (2020) that reported the characterization of *Massilia* spp**.** among the bacterial isolate with plant growth-promoting trait and biocontrol activity isolated from a leguminous plant (*Lotus parviflorus).*

These bacterial isolates characterized in this study was reported to produce IAA which is among the mechanisms employed by PGPR in exerting their positive effect on the plant which corroborates with the previous study (Rana et al. 2011). The elongation of the plant root system for uptake of water and nutrient needed for plant growth is enhanced by the production of IAA by plant growth-promoting rhizobacteria (Egamberdieva et al. 2017). All the bacteria isolates were able to produce ammonia (NH_3_), which corroborates with the finding of Geetha et al. (2014). In the findings, they reported the production of ammonia by rhizobacteria isolated from the green gram rhizosphere. Ammonia production by rhizobacterial was also reported by Kandjimi et al. (2015) to promote plant growth by making nitrogen available for plant growth. Likewise, the production of ammonia by rhizobacteria isolates was reported by Bhattacharyya and Jha (2012) to promote plant growth by preventing the growth of fungal pathogens. In this study, bacterial isolates were all positive to the exopolysaccharide test responsible for their tolerance to different environmental stress. This result corroborates with the finding of Deka et al. (2019) that reported the production of exopolysaccharide by *Bacillus* spp. to be responsible for their acid tolerance and increase the soil aggregate when they are applied to the soil.

All the bacterial isolate characterized in this study were siderophore-producing microbes, which agreed with the previous research study of Shen et al. (2013) they reported that siderophore producing microbes increase plant growth by promoting the acquisition of iron, inhibit the growth of other microorganisms through anabiosis, and inhibit the growth of fungi phytopathogens by limiting the amount of iron need for their development. The isolation and characterization of *Bacillus* spp*.* and *Pseudomonas* spp from various maize rhizosphere in this study have been reported by Figueroa-López et al. (2016) for their plant growth potential, biocontrol activity.

In contrast to the plant growth-promoting characteristic, the biocontrol activity of the bacterial isolates against *Fusarium graminarium* pathogens can be attributed to the production of the lytic enzyme by the bacterial isolates, which disrupts the component of the fungal cell wall, inhibit spore germination and mycelia growth and disrupt protein synthesis (Mardanova et al. 2016).

More so, the antifungal activity of *Bacillus*, *Enterococcus*, and *Pseudomonas* species highlighted in this study have previously been reported to be harmful to fungal pathogens (Brown et al. 2019; Khan et al. 2018; Kurniawan et al. 2018). The plant-growth-promoting and biocontrol activity of *Massilia* spp reported in this study agreed with the study of Araujo et al. (2019). All the bacteria isolate was able to produce catalase which was reported in the previous study to help maintain the ROS level and protect plants against stress (chemical and environmental) (Kumar and Sharma 2012). In the study, all the bacterial isolates showed a different level of tolerance to pH, temperature, salinity, and heavy metal stress. The tolerance of the bacterial isolates characterized in this study to various environmental stress, which could be detrimental to plant health is important for evaluating the effectiveness of the bacterial isolate and the result obtained corroborates with previous findings (Abedinzadeh et al. 2019; Ndeddy Aka and Babalola 2016). Plant growth promotion, photosynthesis, enzyme activity, and protein synthesis are negatively affected by abiotic stress (Enebe and Babalola 2018). Therefore, the co-inoculation of these stress tolerance bacteria strains as an inoculant for plant growth will help the plant to tolerate stress conditions.

Regarding the in vitro potential of the rhizobacterial to enhance maize and soybean seed growth under laboratory condition. Some of the *Bacillus* and *Pseudomonas* species were able to enhance the seed germination effectively than other species. This finding corroborates with the result of Almaghrabi et al. (2014) that reported the effect of *Bacillus* species and *Pseudomonas putida* in enhancing maize seed under laboratory condition. The identification of the functional genes and secondary metabolites that are responsible for plant growth promotion in the genome of the rhizobacterial strains using whole-genome sequencing is an important method to validate in vitro plant growth potential of the rhizobacteria strains, but this a less studied in previous research finding. More so the whole genome sequencing method should be conducted to ascertain if plant growth-promoting genes are present in the bacterial genome. In this study, the rhizobacterial strain possessed plant growth-promoting functional genes in their genome. This agreed with the findings of Crovadore et al. (2020) that reported the whole genome analysis to reveal the plant growth-promoting functional genes in *Pseudomonas* and *Bacillus* species. The production of secondary metabolites by plant growth-promoting rhizobacteria has to be reported to enhance plant growth (Lucke et al. 2020). In this study, the genomic analysis of the rhizobacterial strains revealed production of different secondary metabolites that are responsible for plant growth promotion among which Non-Ribosomal Peptide Synthetase (NRPS), arypolyene, and resorcinol are selected. This study corroborates with the finding of Rieusset et al. (2020) that reported the production of secondary metabolites arypolyene and resorcinol by *Pseudomonas* strains to enhance plant growth. Also, the production of Non-Ribosomal Peptide Synthetase (NRPS) secondary metabolites by *Pseudomonas* and *Bacillus* species had previously been reported (Gu et al. 2020; Martínez-Núñez and y López 2016).

## Conclusion

In the present study, isolation, characterization, stress tolerance, and antifungal activity of PGPR from soybean and maize plant rhizosphere have been studied. Based on the result obtained from this study, the bacteria strains isolated from the rhizosphere of maize and soybeans exhibited more than one plant growth-promoting potentials such as hydrogen cyanide production, IAA production, production of ammonia, antifungal activity, phosphate solubilization, and tolerance to abiotic stress. Furthermore, these microorganisms have shown in vitro antagonistic activity against phytopathogen *Fusarium graminearum,* causing fusariosis in maize, reducing maize production in South Africa, and were able to enhance maize and soybean seed germination under laboratory condition. More so, the whole genome analysis of the rhizobacterial strain performed in this study reveals the presence of functional genes and secondary metabolites responsible for plant growth promotion in the bacteria genome. In evaluating the result obtained from this research study, it can be deduced that there are several plants beneficial bacterial associated with maize and soybeans rhizosphere, and it can be concluded that the plant growth-promoting rhizobacteria characterized in this study possessed biofertilization and biocontrol trait that can make them to be used as bioinoculant for plant growth to ensuring agricultural sustainability and reduce the application of chemical fertilizer which are deleterious to human health and environment.

## Data Availability

All the bacteria strains data characterized using I6S rRNA in this study with their accession number are available in the NCBI database. For the whole genome sequence data: ***Bacillus***** sp. OA1:** The raw reads are available under the Bioproject accession number (PRJNA683742)and Biosample numbers (SAMN17036205). The sequence data obtained in this work have been deposited in the NCBI Sequence Read Archive under accession number (SRX9654894). The genome sequence of *Bacillus* sp. OA1 has been deposited at DDBJ/ENA/GenBank under the accession JAEKPB000000000.1 ***Pseudomonas rhizosphaerae***** OA2:** The raw reads are available under the Bioproject accession number PRJNA683763 and Biosample numbers SAMN17036358. The sequence data obtained in this work have been deposited in the NCBI Sequence Read Archive under accession number SRX9654938. ***Pseudomonas***** sp. OA3:** The raw reads are available under the Bioproject accession number PRJNA683768- *Pseudomonas* sp. OA3)and Biosample number SAMN17036469- *Pseudomonas* sp. OA3). The sequence data obtained in this work have been deposited in the NCBI Sequence Read Archive under accession number SRX9654998- *Pseudomonas* sp. OA3. The genome sequence of *Pseudomonas* sp. OA3 has been deposited at DDBJ/ENA/GenBank under the accession JAEMOM000000000.1.
